# Validity and reliability of electroacoustic probe for diagnosis of developmental dysplasia of the hip

**DOI:** 10.1186/s12887-017-0903-z

**Published:** 2017-06-19

**Authors:** Nicolas Padilla-Raygoza, Georgina Olvera-Villanueva, Silvia del Carmen Delgado-Sandoval, Teodoro Cordova-Fraga, Modesto Antonio Sosa-Aquino, Vicente Beltran-Campos

**Affiliations:** 1 Department of Nursing and Obstetrics, Division of Health Sciences and Engineering, Campus Celaya Salvatierra, University of Guanajuato, Mutualismo 303, 38060 Celaya, Guanajuato México; 2 Department of Physics, Division of Sciences and Engineering, Campus Leon, University of Guanajuato, Lomas del Bosque 103, Leon, 37150 Guanajuato Mexico; 3 Department of Clinical Nursing, Division of Health Sciences and Engineering, Campus Celaya Salvatierra, University of Guanajuato, Av. Ing. Javier Barros Sierra 201, Celaya, 38140 Guanajuato México

**Keywords:** Developmental dysplasia of the hip, Newborns, Sound transmission, Ultrasound Graf technique

## Abstract

**Background:**

Sound transmission is used in the diagnosis of hip dysplasia since the end of the 80’s. Aim of this study is to quantify the validity and reliability of electroacoustic probe for the diagnosis of hip dysplasia in neonates.

**Methods:**

Diagnostic study included neonates aged 4–28 days, whose parents signed an informed consent. The probe was used three times for comparative sound transmission and with extension/flexion; hip ultrasound was performed with Graf technique as gold standard. Kappa was determined for intraobserver and interobserver reliability; validity was calculated with sensitivity, specificity, and predictive values.

**Results:**

100 neonates were included. For the comparative sound transmission, 0.80 and 0.81 Kappa were obtained for the intraobserver and interobserver respectively; with extension/flexion, Kappa 0.98 and 0.95 were obtained for the intraobserver and interobserver respectively. With comparative sound transmission, 44.8%, 97.7%, 76.5% and 91.3% for sensitivity, specificity, positive and negative predictive values, respectively; with extension/flexion test, the sensitivity, specificity, positive and negative predictive values: 82.8%, 99.4%, 96.0%, and 97.1%, respectively.

**Conclusion:**

The electroacoustic probe is moderate valid and reliable for the diagnosis of developmental dysplasia of the hip.

**Trial registration:**

Open Science framework https://osf.io/kpf5s/?view_only=0a9682c6w1c842ad8e1d9a66e8dcf038

## Background

Developmental dysplasia of the hip (DDH) is a range of hip disorders ranging from slight incongruence between the articular surfaces of the ilium and femur to the displacement of the femoral head out of the acetabulum [1]. In Mexico, it is considered that 1% of newborns have hip dysplasia and 75% of macrosomic infants have ultrasound evidence of alterations in the hip; although the evolution of hip dislocation occurs only in 1: 7000 live births [2]. In the United States, DDH is estimated at 1 in 100 infants in the form of instability and in 1 out of every 1000 newborns in the form of hip dislocation [3].

The health professional who takes care of children, is facing a challenge as it is considered, according to Fernandez, that 73% of affected children are diagnosed by parents during the second six months of life [4], although it seems that up to 95% of cases have gone unnoticed by health professionals [1], overshadowing the forecast, and therefore reaching to more invasive treatment and higher chances of disability in the function of the lower extremities.

The clinical diagnosis is made by performing clinical maneuvers such as Ortolani, Barlow, limitation of abduction, asymmetric folds, Galeazzi, and Piston, but these only detect hip subluxation or dislocation [1, 5, 6] and it is intended to achieve an early diagnose using the physical properties of the bone for sound transmission [5–9].

Stone et al., described the use of a tuning fork and stethoscope to diagnose DDH,[7] using comparative test sound transmission (CTST) and comparative sound transmission with extension / flexion (STE/F). Padilla et al., applied both tests in children under 2 years with stethoscope and tuning fork [5], and in neonates with a tuning fork [6, 8], which reported greater validity than the usual clinical maneuvers.

The CTST and STE/F with stethoscope and tuning fork had greater validity than the usual clinical maneuvers [5, 6, 8], but this is a subjective test since a good auditory acuity and good training is required.

**Fig. 1:**
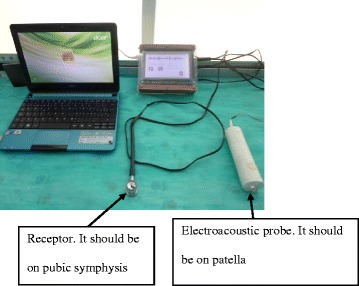
Electroacoustic probe

Padilla et al., evaluated a device based on the transmission of sound with high validity and reliability [9], where the sound wave is propagated through the bone, from the patella to the pubic symphysis, where it is perceived by a receiver which communicates with an amplifier and converts the sound received into digits and subsequently confirming the diagnosis with an ultrasonography hips. With results previously reported by Padilla et al., in 2014 [9], an electroacoustic probe was designed, this is an electromagnetic device that is capable of producing sinusoidal signals at a frequency that is required in a range of 1 Hz to 400 kHz and can be an integer or decimal. It also includes a sound wave generated by an electroacoustic amplifier pitch; this is transmitted through the lower end of the patella to the pubic symphysis. A touch screen shows the sound transmission recorded in graphic or digital form. The receiver of the electroacoustic probe is placed on the pubic symphysis and the perceived sound wave is transformed which is then presented in decibels on the screen of the device, allowing to perform similar tests to the tuning fork and stethoscope tests, with the difference of this being an objective test (Fig. 1).

With sound transmission tests, tuning fork and stethoscope, bone radar (Mexican Pat. N°337,887) or electroacoustic probe, for healthy hips the transmission sound is the same and equal in both hips, but if there is an immature or dysplasic hip, the sound is lower because the contact between components of the hip is minor; with the sound transmission test with extension/flexion, when the extremity is flexed the contact between components of the hip is higher if the hip is immature or dysplasic, and the sound transmission increases compared with flexion.

The aim of the study was to identify the reliability and validity of the electroacoustic probe for DDH diagnosis in neonates of Celaya, Guanajuato.

## Methods

The protocol was approved by the Bioethics Committee of the Division of Health Sciences and Engineering Campus Celaya Salvatierra, University of Guanajuato.

It is a diagnostic test study based on the community. It was held in Celaya, Guanajuato between January and December 2014.

Mothers of newborns from three public and four private hospitals of the city, were invited to participate in the study, performing it on the facilities of the University of Guanajuato.

### Selection of participants

#### Inclusion criteria

Neonates of 4–28 days old, whose parents agreed in writing that their child may participate.

#### Exclusion criteria

Newborns with rigid, embryological hip dislocation.

#### Variables

Gender, age, area of ​​residence, birth weight and height, weight and height when beginning study were measured.

The CTST and STE/F were applied with the electroacoustic probe. For CTST, the newborn was placed supine with legs extended; the electroacoustic tuning fork was placed on the left kneecap and the receiver on the pubic symphysis; on the digital display of the amplifier “on” is pressed and the sound transmission lasts 5 s, whose wave is picked up by the receiver and the results in decibels appear on the screen. The electroacoustic tuning fork is placed on the opposite patella and the same procedure was performed. If the sound is lower in any of the sides, this is an indicative of an alteration in the hip.

For STE/F, the newborn was placed in a supine position with the pelvic limbs extended. The electroacoustic tuning fork is placed on the left kneecap and the receiver on the pubic symphysis, pressing “on” generates a sound wave for 5 s, which is picked up by the receiver and the results in decibels are shown on the screen. The hip is flexed at 90 ° and the measurement procedure is repeated. If the sound increases while bending, this indicates an altered hip (Fig. 1).

The newborns subsequently underwent hip ultrasonography using Graf’s method, which was applied with a portable ultrasound transducer, Honda MS2000. The static and dynamic tests were performed and the angles alpha and beta were measured for both tests on both hips. The following criteria was taken for the diagnosis of DDH [10, 11]:

I Graf angle α > 60 ° and angle β <55 °, healthy hip

Graf II 44–59 ° angle α and angle β 55 ° -77, physiological immaturity

Graf III and IV angle α <43 ° and β angle > 77 °, subluxation or dislocation.

#### Procedures

Invitations to participate were distributed to parents in vaccination units; in private hospitals, mothers who gave birth were also invited. Those who attended the University of Guanajuato, were given an information sheet for parents, and the formulated questions were answered. Later they were asked to sign an informed consent. Those neonates who made it had their height and weight measured and their parents were asked about the date of birth, gender, birth height and weight, and area of ​​residence. Thereafter the CTST and STE/F tests were applied to the newborns, twice with the electroacoustic probe by an investigator and a third time by a different investigator. The newborn immediately underwent hip ultrasound using Graf’s technique; the ultrasonographer was blinded to the results of the sound tests.

#### Sample size

Expecting a sensitivity of 85% with a prevalence for DDH of 10%, the minimum sample size is 11 neonates with a 95% of precision and 90% of power (3.1 Epidat, 2005, Xunta de Galicia and PAHO)

#### Statistic analysis

Descriptive statistics were used for the study variables.

For intraobserver reliability, Kappa was calculated by comparing measurements 1 and 2 and interobserver Kappa by comparing measurements 1 and 3.

For the validity, the sensitivity, specificity, and predictive values were calculated for the test of sound transmission with electroacoustic probe using Graf hip ultrasound technique as gold standard.

The statistical analysis was performed in 13.0® STATA (Stata Corp., College Station, TX, USA).

## Results

The sample consisted of 200 hips from 100 newborns. Female neonates predominated (64%), newborns living in urban areas (72%), 16% reported having a family history of DDH, parents being the most frequently reported as affected (4%) and 9% other relatives (cousins, uncles or grandparents) (Table 1).

**Table 1:** Sociodemographics categorical characteristics of newborns, Celaya, 2014 (*n* = 100)

Variable	Frequency	
	n	%
Gender		
Male	36	36.0
Female	64	64.0
Residence area		
Urban	72	72.0
Sub urban	19	19.0
Rural	9	9.0
Family background of DDH		
Yes	16	16.0
No	84	84.0
Who is affected		
Parents	4	4.0
Sibilings	3	3.0
Others	9	9.0
No one	84	84.0

Source: Questionnaire of the study

*DDH* Developmental dysplasia of the hip

The quantitative characteristics of the infants were: age range 4–28, average 14.7 ± 7.9 days; birth weight was 1.9 to 4.2 with a mean of 3160.3 ± 426.9 g; height at birth was 44–55 with an average of 49.8 ± 2.1 cm; weight at the beginning of the study was 2270–5100 with an average of 3538.7 ± 586.3 g; height at the beginning of study was 47–60 with an average of 51.9 ± 2.7 cm.

Reliability for CTST and STE/F is shown in Table 2, excellent intraobserver and interobserver reliability was found for both tests.

**Table 2:** Reliability intra and inter-observer for the electroacoustic probe, Celaya, 2014 (*n* = 200)

	Second measure		Third measure	
	+	-	+	-
Comparative test sound transmission				
First measure				
+	12	4	13	4
-	1	183	1	182
Kappa (95%CI)	0.80 (0.63–0.97)		0.81 (0.65–0.97)	
Sound transmission with extension/flexion				
First measure				
+	24	1	24	1
-	0	175	1	174
Kappa (95%CI)	0.98 (0.93–1.0)		0.95 (0.89–1.0)	

Source: Measures of the study with electroacoustic probe

The validity is shown for the three measurements for CST in Table 3; no significant differences were found. The sensitivity is low due to the bilateral affectations causing false negative results to be given.

**Table 3:** Validity of comparative test sound transmission, Celaya, 2014(*n* = 200)

		Ultrasonography					
		+	-	Sensitivity % (5%CI)	Specificity % (95%CI)	Predictive value + % (95%CI)	Predictive value – % (95%CI)
First measure	CT			44.83 (25.00–64.65)	97.66 (95.10–100.00)	76.47 (53.37–99.58)	91.26 (86.89–95.62)
	ST + -	13 16	4 167				
Second measure	CT			37.93 (18.55–57.31)	98.83 (96.93–100.00)	84.62 (61.16–100.00)	90.37 (85.88–94.87)
	ST + -	11 18	2 169				
Third measure	CT			37.93 (18.55–57.31)	97.66 (95.10–100.00)	73.33 (47.62–99.05)	90.27 (85.73–94.81)
	ST + -	11 18	4 167				

*CTST* Comparative test sound transmission 95%CI = 95% confidence intervals

Source: Measurements in the study with electroacoustic probe and ultrasonography of the hips

The validity test for STE/F is shown in Table 4 for the three measurements. The sensitivity, specificity and predictive values are higher than with the CTST because each hip is evaluated separately diagnosing more accurately the bilateral cases; no significant differences were found between the three measurements for the different parameters of validity.

**Table 4:** Validity of sound transmission with extension/flexion, Celaya, 2014 (*n* = 200)

		Ultrasonograhy					
		+	-	Sensitivity % (95%CI)	Specificity % (95%CI)	Predictive value + % (95%CI)	Predictive value – % (95%CI)
First measure	ST E/F			82.76 (67.29–98.23)	99.42 (97.98–100.0)	96.00 (86.32–100.0)	97.14 (94.39–99.90)
	+ -	24 5	1 170				
Second measure	ST E/F			82.76 (67.29–98.23)	100.0 (99.71–100.0)	100.0 (97.92–100.00)	97.16 (94.42–99.90)
	+ -	24 5	0 171				
Third measure	ST E/F			86.21 (71.93–100.0)	100.0 (999.71–100.0)	100.0 (98.00–100.0)	97.71 (95.21–100.0)
	+ -	25 4	0 171				

*STE/F* Sound transmission with extension/flexion

*95%CI* 95% confidence intervals

Source: Measurements in the study with electroacoustic probe and ultrasonography of the hips

From the ultrasound employing the Graf method: for the left side, 85 healthy hips (Graf 1), 13 hips with physiological immaturity (Graf 2) and 2 hips with subluxation (Graf 3), were obtained; for right hip, 86 healthy hips (Graf 1) and 14 hips with physiological immaturity (Graf 2) were diagnosed.

## Discussion

The sample was not representative of the infant population since the participation was by invitation and voluntarily, preventing the generalization of the results, which is a major drawback of the study.

There were no infants excluded as no rigid dislocations were detected.

The frequency of family history was high (16%) (Table 1). There may be a bias of the subject, for if they had relatives who had suffered DDH, they might have agreed to participate more easily, compared to those without such a history.

No diagnosis of complete hip dislocation was obtained.

Other disadvantages of the study is that only 2 subluxation cases were diagnosed and that physiologically immature hips can evolve mainly from healthy hips and/or from a small percentage subluxation.

Intraobserver and interobserver reliability of electroacoustic probe (Table 4) was higher than those reported by the bone radar [9].

The CTST shows a low sensitivity, because bilateral cases are detected as false negatives, from 37.9% to 44.8% in the three measurements; these are similar results to those reported by Padilla et., in 1996, of 27.27% with CTST in neonates using the tuning fork and stethoscope [6], and with the radar bone (Mexican Pat. N° 337,887) device a greater sensitivity of 60.9%^9^ was found because less bilateral cases were detected, but the specificity showed values ​​above 90%, a positive predictive value greater than 73%, and a negative predictive value greater than 90% (Table 3); similar results were reported with tuning fork and stethoscope [6, 8], and with the radar bone (Mexican Pat. N° 337,887) [9].

For the STE/F, validity was raised with percentages above 80% for sensitivity, and 90% for specificity and predictive values ​​(Table 4); similar but slightly lower results reported by Padilla et al. using the radar bone (Patent Pending, University of Guanajuato) [9] and similar to those reported by Padilla et al. using the tuning fork and stethoscope [6, 8].

Kwong et al., [12] designed a device for measuring the difference of sound transmission in the hip and found a sensitivity of 100% and specificity of 75% with cutoff points with a 2 dB difference. Celaya results show lower sensitivity (90%) and higher specificity (100%) (Tables 3 and 4).

The Primary Care Physicians can use the electroaccoustic device as inexpensive screening tool to detect alterations in the hip, and it complement physical exam of neonates.

## Conclusions

The use of electroacoustic probe showed moderate sensitivity and high specificity, and high repeatability in immaturity hip.

It is needed further research in a population with more cases of DDH to demonstrate if the electroacoustic probe have higher sensitivity.

An advantage of the electroacoustic probe is that it detects from physiological immaturity to hip subluxation, as reported in the study.

## Abbreviations

CTST: Comparative test sound transmission
DDH: Developmental dysplasia of the hip
STE/F: Spund transmission with extension/flexion
